# Substandard Antimalarials Available in Afghanistan: A Case for Assessing the Quality of Drugs in Resource Poor Settings

**DOI:** 10.4269/ajtmh.14-0394

**Published:** 2015-06-03

**Authors:** Mirza Lalani, Harparkash Kaur, Nader Mohammed, Naiela Mailk, Albert van Wyk, Sakhi Jan, Rishtya Meena Kakar, Mohammed Khalid Mojadidi, Toby Leslie

**Affiliations:** London School of Hygiene & Tropical Medicine, London, United Kingdom; Health Protection and Research Organisation, Kabul, Afghanistan; HealthNet TPO, Kabul, Afghanistan

## Abstract

Good-quality antimalarials are crucial for the effective treatment and control of malaria. A total of 7,740 individual and packaged tablets, ampoules, and syrups were obtained from 60 randomly selected public (*N* = 35) and private outlets (*N* = 25) in Afghanistan. Of these, 134 samples were screened using the Global Pharma Health Fund (GPHF) MiniLab^®^ in Kabul with 33/126 (26%) samples failing the MiniLab^®^ disintegration test. The quality of a subsample (*N* = 37) of cholorquine, quinine, and sulfadoxine/pyrimethamine tablets was assessed by in vitro dissolution testing following U.S. Pharmacopeia (USP) monographs at a bioanalytical laboratory in London, United Kingdom. Overall, 12/32 (32%) samples of sulfadoxine/pyrimethamine and quinine were found not to comply with the USP tolerance limits. Substandard antimalarials were available in Afghanistan demonstrating that continuous monitoring of drug quality is warranted. However, in Afghanistan as in many low-income countries, capacity to determine and monitor drug quality using methods such as dissolution testing needs to be established to empower national authorities to take appropriate action in setting up legislation and regulation.

## Background

Poor-quality drugs† †The term “poor-quality drugs” for the purposes of this study encompasses SFFC,^4^ (spurious, falsely labeled, falsified, counterfeit), substandard, and degraded drugs. in malaria-endemic countries are a threat to effective disease control and there has been an increase in reports of their detection in developing countries.^1^,^2^

Drug quality reports are lacking for 63 (60.3%) of 104 malaria-endemic countries including Afghanistan. A recent review found that 30% (2,813) of a total of 9,348 antimalarial drug samples from parts of Asia, central and south America, and sub-Saharan Africa had failed chemical content/packaging analysis.^3^

In resource-constrained countries with the lack of an effective drug regulatory system and drug testing facilities, poor-quality antimalarial drugs may be widely available.^4^ Afghanistan is one such country, which lacks both drug legislation and regulation, limited infrastructure for conducting assessment of drug quality,^5^ and long, porous borders with six different nations.

The population of Afghanistan was 29,790,000, in 2011, with an estimated 60% at risk of malaria.^6^,^7^ Malaria is endemic throughout Afghanistan, at altitudes below 2,000 m, especially in the populous rice-growing regions in the eastern and north-eastern part of the country.^8^ According to World Health Organization (WHO) estimates, there were 1,934 (per 100,000) cases of malaria in Afghanistan in 2011.^9^ *Plasmodium vivax* infection is predominant, accounting for 80–90% of malaria cases annually with the remainder caused by *P. falciparum* species.^10^ Chloroquine remains the most effective antimalarial drug treatment of *P. vivax* and artemisinin combination therapy (ACT) is recommended for treating uncomplicated *P. falciparum* and mixed infections.^11^

In recent years, malaria control activities have relied on mass distribution of long-lasting insecticide-treated nets.^12^ This approach, coupled with a slowly improving post-conflict health services package, has succeeded in reducing malaria transmission. However, challenges remain with efforts undermined by a lack of infrastructure and extreme poverty.^13^ A key facet of post-conflict development for nongovernmental organizations (NGOs) in Afghanistan was the basic package of health service (BPHS).^14^ A component of the BPHS is the provision of medicines through public health sector clinics which relies on funding from international donors and partners such as the U. S. Agency for International Development (USAID), the World Bank, and the European Commission and is implemented by NGOs operating under the stewardship of the Ministry of Public Health (MoPH).^15^ Afghanistan has no formal pharmaceutical industry and all drugs, including antimalarials, are imported with the private sector thought to have over 200 importers.

Evidence of poor-quality antimalarial tablets originated from southeast Asia where “counterfeit or fake”^16^ antimalarials were found with very sophisticated packaging that mimicked an existing brand but did not contain the stated active pharmaceutical ingredient (API).^17^,^18^ In addition, in Afghan refugee camps on the Pakistan/Afghanistan border, an epidemic of malaria may have led to a spurious conclusion of sulfadoxine/pyrimethamine resistance^19^ had the drugs not been analyzed and found to be “substandard.”^20^ Another study conducted in a region in Yemen found substandard samples of sulfadoxine/pyrimethamine and chlorquine analyzed by content and dissolution testing that were collected at various levels of the distribution chain from the public and private sector.^21^

To date, there is a lack of published research findings on the quality of locally available antimalarial drugs in west Asia including Afghanistan where the market for antimalarials is substantial. The aim of this study was to identify the range of drugs available on the market and the quality of antimalarial drugs in the public and private outlets in urban and rural locations in Afghanistan.

## Methods and Materials

### Study location and sample collection.

**Figure 1.**
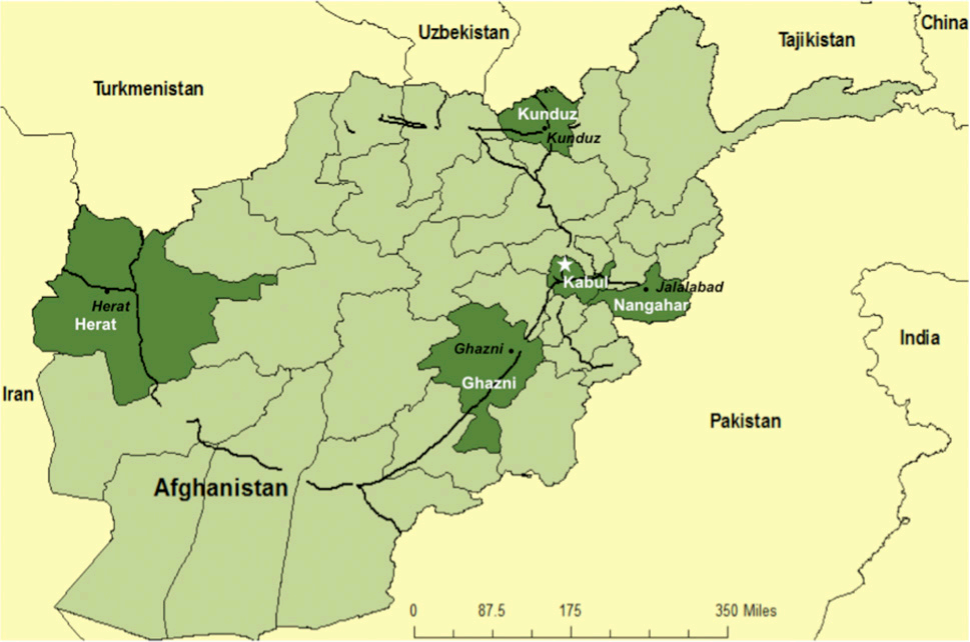
Afghanistan provinces where antimalarial drugs sampled for quality, 2009. The darker green areas represent the five study regions. The thicker black lines represent the primary roads in Afghanistan.

The study was conducted in 5 out of a total of 33 provinces in Afghanistan (Figure 1). These were chosen as they contain the main trading hubs with neighboring countries and are thus the major import and transit points for imported pharmaceuticals.

Samples were obtained in August 2009, to coincide with the peak malaria season, from 60 drug collection points across the 5 study provinces. This included public and private providers and the informal market (outlets not registered with the MoPH). In each of the five selected study provinces one rural district was randomly selected and the provincial capital city was also surveyed. Private sector outlets were sampled because they were concentrated in locations around urban centers. Private provider drug collection points consisted of markets, street vendors, shops, private pharmacies, and not-for-profit NGO pharmacies in clinics. Public providers included community health workers, pharmacies within government-run clinics and hospitals. Data were collected on the type, name, and location of facility, sampled in the selected area.

### Study procedure.

Fieldwork was conducted in two stages. The first stage involved establishing the sampling frame. Two members of the project team compiled a comprehensive list of drug outlets within the selected city and one randomly selected rural district within the province. A census was conducted for private sector outlets to provide a complete list. The public sector outlets were randomly selected from a preexisting list of clinics in each district. The comprehensive combined list was then used to randomly select five private sector outlets in the urban area and seven public sector outlets in the rural area. The survey started at a central point and used a systematic sampling interval (four or five) to identify the private sector sampling points for inclusion if they were on the census list.

The second stage was concentrated on the sampling of antimalarial drugs. Drug samples and associated informations were gathered by collectors who visited the private drug outlets identified in Stage 1. The collector went to the outlet posing as a normal costumer (covert collection) and purchased the drugs. In public sector outlets (pharmacies within clinics and hospitals run by the government), drugs were obtained without any payment in an overt way. The drug collector requested samples from the pharmacist or doctor, explaining the study and providing an authority letter from the MoPH. The samples consisted of all available antimalarial tablets, injections (ampoules), and suspensions (syrups). At each site, five adult doses of each available antimalarial drug were obtained, in their original packaging, and stored in ziplock bags marked with the facility code. On the day of obtaining the samples, a drug collection sheet was used to record information on the date, place, and conditions of purchase (name of the drug indicated by the vendor, name stated on the product, and the price). They were stored in a dark, dry, and air-conditioned room (at 22°C) before being transported to the laboratory in Kabul for assessment within a month of sampling.

Initial screening for the quality of 134 drug samples was performed using a Global Pharma Health Fund (GPHF) MiniLab^®^ (GPHF, Frankfurt, Germany) at the HealthNet TPO office in Kabul.^22^ A subset of 40 samples were analyzed by in vitro dissolution methods and content analysis of the API by high-performance liquid chromatography with ultraviolet diode array detection (HPLC-UV-PDA) following previously described standard operating procedures (SOPs) used to determine the quality of drugs.^23^ This work was undertaken in a bioanalytical laboratory at the London School of Hygiene and Tropical Medicine in November 2009.

### Drug quality screening test (GPHF MiniLab^®^).

One sample from each collected drug (four samples were stored for possible additional analysis) was screened. Two physical testing methods (visual inspection and disintegration testing) and a chemical method (semiquantitative thin-layer chromatography [TLC]) were performed and drugs were classified as a pass or fail as per the SOPs outlined in the MiniLab^®^ manual.^24^

### Drug quality by content analysis (HPLC-UV-PDA).

Content analysis was carried out on 40 samples using a Dionex Ultimate 3000 HPLC system (Thermofisher, Hemel Hempstead, United Kingdom). The amount (mg/mL) of API was determined from the calibration curve for each API generated, using pure compounds purchased from Sigma Aldrich, United Kingdom.^25^

Quality of artesunate samples was determined by dissolving the tablet in methanol to produce a 2.5 mg/mL solution and injecting it onto the HPLC column. Chloroquine, sulfadoxine/pyrimethamine, and quinine sample tablets were dissolved in methanol to produce a 0.6 mg/mL solution. The USP rules as outlined in USP 24^26^ for content analysis stipulate that for each tablet of sulfadoxine/pyrimethamine and quinine 90–110% of the stated API should be measured. For chloroquine, it should be 93–107% and for artesunate 95–105% of the stated API.

### Drug quality by dissolution analyses.

Dissolution analysis was performed using the Pharma Test PT 017 dissolution apparatus (Pharma Test Group, Pharma Test, Hainburg, Germany). Quality of the formulations of sulfadoxine/pyrimethamine, quinine, and chloroquine (*N* = 37) was determined using the in vitro dissolution testing protocols as detailed in the drug monographs outlined in USP 24. Classification of analyzed samples was as per the criteria in Table 1 for the quality of the drugs.

### Ethical aspects.

Ethical approval was given by the Institutional Review Board of the Ministry of Public Health, Afghanistan.

## Results

### Survey of facilities.

A total of 60 drug outlets were surveyed, 35 from the public sector and 25 from the private sector. Although no informal sector outlets were identified in the urban study area, it is recognized that informal drug sellers probably exist in the provinces surveyed.

### Drugs collected.

A total of 7,740 individual and packaged antimalarial tablets, ampoules, and syrups were obtained from the private (*N* = 3,548, 46%) and public sectors (*N* = 4,192, 54%) (Table 2). The private sector had a greater range of drugs available than the public sector. Chloroquine (*N* = 3,973, 51%), quinine (*N* = 2,446, 32%), and sulfadoxine/pyrimethamine (*N* = 601, 8%) were the most abundant drugs and were available in both sectors. The combination treatment, artesunate and sulfadoxine/pyrimethamine was available in 46% (16 out of 35) public sector clinics but not in the private sector. A total of six private sector outlets in three provinces were found to stock halofantrine whereas amodiaquine was also obtained in four private sector outlets in Nangahar and Ghanzi provinces, even though neither of these drugs is listed in the national guidelines.

Artesunate and artemether tablets as monotherapy are not licensed for use in Afghanistan. Artesunate tablets were available in one private sector outlet in Kabul and one public sector outlet in Herat. Artemether tablets were also purchased from the private sector in Nangahar and Ghazni. Primaquine was only available in one private sector outlet in Kabul.

### GPHF MiniLab^®^ drug screening.

Overall none of the drugs failed visual inspection or the TLC testing following the MiniLab guidelines.

Visual inspection entailed examining packets and tablets for obvious discoloration or other defects and none were found. Drug packaging appeared appropriate with correctly stated dose, type of drug, batch number, expiry date, and manufacture date as per MiniLab^®^ requirements for visual inspection. However, original drug packaging from manufacturers was not available for comparison. It was also found that 9% (*N* = 12) of the 134 drug packages that were examined contained the instruction leaflet (either on the packaging or the package insert) in Pashto or Dari—the most commonly spoken official languages of Afghanistan. All others were in foreign languages including, Urdu, Hindi, German, English, or French.

However, a few samples of chloroquine (33%; *N* = 25 out of 77 tablets) and quinine (37.5%; *N* = 8 out of 22 tablets) samples failed the disintegration test. A total of 33 out of the 126 (26%) samples failed the disintegration test (Table 3).

### Dissolution and Content analysis.

Of the 134 samples screened by the MiniLab^®^, a subset of 40 were subjected to dissolution and content analysis by HPLC (Table 4). These 40 samples composed of all of the failing chloroquine tablets (*N* = 25) and three out of eight of the failing quinine tablets as well as nine sulfadoxine/pyrimethamine tablets and three artesunate tablets.‡ ‡The nine sulfadoxine/pyrimethamine and three artesunate samples were selected as they had a sufficient number of additional tablets (remaining following the MiniLab screening) to be analyzed by dissolution and HPLC. These 12 samples all passed the MiniLab tests.

Poor manufacturing practices, drug degradation, and the use of incorrect excipients will lead to poor in vitro dissolution profiles and low API resulting in compromised bioavailability and a poor-quality drug. Of 37 samples (chloroquine *N* = 25; sulfadoxine/pyrimethamine *N* = 9; quinine *N* = 3) tested using the dissolution analysis to determine the quality of the formulations, 12 (32%; *N* = 9 sulfadoxine/pyrimethamine and quinine *N* = 3) did not meet the set USP tolerance limits and were therefore of poor quality. All of the cholorquine (*N* = 25) samples tested by dissolution met the USP tolerance limits for good quality.

The content analysis by HPLC of all samples found that they contained the stated amounts of active ingredients.

All of the drugs were within their expiry date at the time of analysis and of the 40 drugs tested for API content and dissolution, 35/40 (88%) were manufactured in Pakistan. The remaining five samples were manufactured in Iran (*N* = 2) and India (*N* = 3).

## Discussion

The antimalarials quinine and sulfadoxine/pyrimethamine did not meet USP tolerance limits for dissolution in this study. Nevertheless, they passed visual inspection of the packaging (as per MiniLab^®^ requirements) and were complaint with HPLC content analysis (contained sufficient API). They should therefore be classified as substandard drugs according to the WHO definition.§ §Substandard medicines (also called out of specification [OOS] products) are genuine medicines produced by manufacturers authorized by the National Medicines Regulatory Authority (NMRA) which do not meet quality specifications set for them by national standards.

None of the antimalarials analyzed in this study contained low APIs. Most studies that have detected drugs with low APIs will refer to them as substandard drugs.^27^ However, this approach is simplistic because poor dissolution can reduce the bioavailability of drugs and so also needs to be considered. The public health implications of antimalarials with poor bioavailability are unknown but substandard drugs can lead to poor treatment outcomes, wasted financial resources by prolonging illnesses, increase the potential of recrudescence, and may propagate the development of drug resistance.^28^

The noncompliance by dissolution of quinine and sulfadoxine/pyrimethamine may be a result of being manufactured at a facility without good manufacturing practice (GMP) and corroborates evidence from other studies.^29^,^30^ Storage conditions at the time of collection were not comprehensively recorded. Inadequate storage of drugs may cause degradation reducing the API content^31^ due to extremes in temperature and humidity. However, no known degradation products have been reported for these drugs.

Sulfadoxine/pyrimethamine resistance is widespread in many countries in southeast Asia and sub-Saharan Africa.^32^ Substandard sulfadoxine/pyrimethamine, such as those detected in this study, may increase the risk of *P. falciparum* resistance in Afghanistan, with the consequence that when sulfadoxine/pyrimethamine and artesunate are given in combination this may effectively amount to artesunate monotherapy. Moreover, quinine is currently indicated in Afghanistan alongside injectable artemether for severe malaria. The administration of substandard quinine such as those found in this study may lead to delay in recovery or, worse still, treatment failure.

At the time of sampling, national antimalarial treatment guidelines in Afghanistan recommended chloroquine as the first-line treatment of *P. vivax* (alongside a 14-day course of primaquine, where the G6PD status of the patient can be ascertained) as well as for cases of unconfirmed malaria. Indeed, chloroquine, sulfadoxine/pyrimethamine, and quinine were by far the most frequently stocked antimalarials in the outlets comprising 91% of the total individual tablets, ampoules, and syrups that we obtained. The abundance of quinine and sulfadoxine/pyrimethamine in both sectors is discordant with national treatment guidelines as neither drug (as a monotherapy) is regarded as a first-line malaria treatment of uncomplicated *P. falciparum* or *P. vivax* malaria.^33^ Artesunate combined with sulfadoxine/pyrimethamine as combination therapy is used for confirmed *P. falciparum* malaria cases and mixed infections and yet it was unavailable in most of the public sector clinics and all of the private sector outlets sampled.^34^

This study found other antimalarials available that are not recommended by national treatment guidelines including oral artemisinin monotherapy, which was recommended for withdrawal from sale worldwide by the WHO in 2006.^35^ Unfortunately, despite this WHO mandate, a few countries and manufacturers still permit marketing of oral artemisisnin monotherapies.^36^,^37^ Use of monotherapy has been proposed as a potential factor for increasingly slow parasite clearance times with the artemisinins in southeast Asia.^38^ Other antimalarials such as amodiaquine and halofantrine were both available in the private sector. Amodiaquine has been shown to be largely ineffective against *P. falciparum* in this region^39^,^40^ and halofantrine use has been associated with cardiotoxicity and therefore its therapeutic use is restricted to instances in which safer alternative drugs are not available.^41^

In Afghanistan, around 50% of the population seeks treatment of malaria from the private sector, thus reinforcing the need to ensure that only antimalarial drugs that are recommended by national treatment guidelines are available and that they are of good quality (Leslie T and others, unpublished data).

In this study, 74% of the tablets (93/126) passed the MiniLab disintegration test, 26% consisting of 25 tablets of chloroquine and 8 tablets of quinine did not. However, all samples did pass the visual inspection and TLC tests. The results indicate some disparity when comparing MiniLab^®^ disintegration findings and the dissolution testing results with the chloroquine and sulfadoxine/pyrimethamine samples. Our findings are similar to previous results where the MiniLab^®^ disintegration test has demonstrated a low sensitivity to detect noncompliance in the dissolution method for samples of sulfadoxine/pyrimethamine.^42^ The disintegration of drugs as per MiniLab^®^ guidelines may not be a clear indicator for bioavailability. However, reduced disintegration may indicate a difference in a drug's bioavailability. Screening tests such as the MiniLab^®^ are relatively inexpensive, rapid, and provide a simple assessment of drug quality and therefore have an important role to play in the monitoring of drug quality in resource-poor and rural settings such as in Afghanistan. However, the MiniLab^®^ cannot be relied on unequivocally for drug quality monitoring systems and to obtain definitive results, precise analytical methods such as HPLC and dissolution are required.^43^

The majority of drug packaging and patient information leaflets (PILs) were found to be printed in non-local languages. This may be a factor for poor adherence to the treatment as patients may not understand how to take the drug. Afghanistan does not manufacture its own antimalarials, hence, provision with packaging and PILs in local languages will be difficult. Antimalarials with pictograms for dosing could be an alternative, as this has been shown to be acceptable to patients and may improve adherence.^44^

The findings of this investigation suggest that, good-quality antimalarials were available in the public and private sectors (particularly for chloroquine and artesunate) in Afghanistan. However, the presence of substandard quinine and sulfadoxine/pyrimethamine warrants further investigation to check for prevalence of poor-quality antimalarials. In addition, health facilities need to improve storage for drugs, even though this is often challenging in hot and humid climates with intermittent power supply.

This study was limited by the sample size and the lack of samples obtained from the informal sector. Drug analysis using “gold standard” methods such as HPLC and dissolution testing need to be undertaken with a larger set of samples than the number tested here to obtain a prevalence estimate of antimalarial drug quality. In addition, sampling of the informal sectors such as mobile vendors and itinerant drug sellers is required, given this sector, in most malaria-endemic countries is unregulated and unquantified but highly accessed for the purchasing of antimalarials by a notable proportion of the population, especially in rural areas.^45^

In conclusion, all antimalarials analyzed by HPLC content analysis were found to be compliant with USP tolerance limits. However, antimalarials with poor dissolution profiles were detected and such drugs should also be acknowledged as substandard.

The discovery of substandard antimalarials and the availability of drugs that are outside of national policy and guidelines warrant an effective drug quality surveillance system and more stringent antimalarial drug regulation in Afghanistan. Allocating adequate funds to monitor the quality of antimalarials drugs should yield positive outcomes for public health.

Increased monitoring of antimalarial drug quality through routine surveillance with activities focused on the most “at risk areas,” for example, main routes for imported drugs, is required. Capacity building for sophisticated drug quality testing facilities as part of an integrated drug quality surveillance system is necessary. In addition, improvement of legislation for ensuring that package information is either available in local languages or in a format that would be deemed acceptable to the local population is needed. Furthermore, there must be an increase in efforts to engage and educate the private and public sectors to comply with national policy guidelines relating to the appropriate use of antimalarial drugs.

## Figures and Tables

**Table 1:** Classification for quality analysis by dissolution of chloroquine, sulfadoxine/pyrimethamine, and quinine

Drug	Good quality
Chloroquine	> 0.278 mg/mL at 30 minutes (250 mg dose)
	> 0.167 mg/mL at 30 minutes (150 mg dose)
Sulfadoxine	> 0.3 mg/mL at 30 minutes (500 mg dose)
Pyrimethamine	> 0.015 mg/mL at 30 minutes (25 mg dose)
Quinine	> 0.250 mg/mL at 30 minutes (300 mg dose)

**Table 2:** Total number of individual antimalarial tablets, ampoules, and syrups obtained, by province and sector

Drugs	Kabul		Nangahar		Herat		Kunduz		Ghazni		Total		
	Private, *N* = 5	Public, *N* = 7	Private, *N* = 5	Public, *N* = 7	Private, *N* = 5	Public, *N* = 7	Private, *N* = 5	Public, *N* = 7	Private, *N* = 5	Public, *N* = 7	Private (%), *N* = 25	Public (%), *N* = 35	Grand total (%), *N* = 60
Chloroquine tablets	300 [4]*	230 [7]	450 [5]	425 [7]	320 [4]	410 [6]	550 [5]	500 [7]	290 [4]	250 [4]	1,910 (51%)	1,815 (49%)	3,725 (48%)
Chloroquine syrup	16 [4]	14 [2]	29 [5]	31 [6]	3 [1]	9 [3]	15 [2]	20 [3]	2 [1]	11 [2]	65 (43%)	85 (57%)	150 (2%)
Chloroquine Injection	0	0	6 [1]	68 [4]	24 [1]	0	0	0	0	0	30 (31%)	68 (69%)	98 (1%)
Fansidar tablets (sulfadoxine/pyrimethamine)	65 [4]	50 [3]	45 [2]	90 [6]	20 [1]	48 [4]	69 [4]	49 [3]	95 [4]	60 [3]	294 (49%)	297 (51%)	591(8%)
Fansidar syrup	0	0	5 [1]	0	0	25 [1]	5 [1]	5 [1]	0	0	10 (100%)	0	10 (0.2%)
Halofantrine tablets	0	0	12 [2]	0	0	0	66 [3]	0	24 [1]	0	102 (100%)	0	102 (1%)
Amodiaquine tablets	0	0	140 [3]	0	0	0	0	0	50 [1]	0	190 (190%)	0	190 (2%)
Amodaquine syrup	0	0	13 [3]	5 [1]	0	0	0	0	0	0	13 (72%)	5 (18%)	18 (0.2%)
Quinine tablets	200 [1]	550 [3]	328 [3]	150 [1]	240 [2]	284 [3]	0	400 [2]	0	185 [2]	568 (27%)	1,569 (73%)	2,137 (28%)
Quinine injections	90 [2]	0	8 [1]	54 [2]	0	42 [1]	0	100 [2]	0	0	98 (32%)	211 (68%)	309 (4%)
Artesunate + sulfadoxine/pyrimethamine tablets	0	16 [4]	0	15 [5]	0	9 [3]	0	8 [2]	0	11 [2]	0	59 (100%)	59 (0.7%)
Artesunate tablets	56 [1]	0	0	48 [1]	0	0	0	0	0	0	56 (54%)	48 (46%)	104 (1%)
Artemether tablets	18 [1]	15 [1]	64 [1]	0	0	5 [1]	0	0	72 [1]	0	136 (100%)	0	136 (1%)
Artemether syrup	0	0	5 [1]	0	3 [1]	0	0	0	0	0	8 (100%)	0	8 (0.1%)
Artemether injections	0	0	0	8 [1]	0	10 [2]	0	0	0	25 [1]	18 (34%)	35 (66%)	53 (0.5%)
Primaquine tablets	50 [1]	0	0	0	0	0	0	0	0	0	50 (100%)	0	50 (0.5%)
Total	795	875	1,105	894	610	842	705	1,082	533	542	3,548 (46%)	4,192 (54%)	7,740

* Number of facilities that stock each drug.

**Table 3:** Outcome of the MiniLab^®^ testing methods

Drug	Number (samples*) tested	Visual inspection		TLC		Disintegration test†	
		Pass	Fail	Pass	Fail	Pass	Fail
Chloroquine tablets	77	77 (100%)	0	77 (100%)	0	52 (67%)	25 (33%)
Sulfadoxine/ Pyrimethamine tablets	12	12 (100%)	0	12 (100%)	0	12 (100%)	0
Artesunate tablets	14	14 (100%)	0	14 (100%)	0	14 (100%)	0
Artemether syrup	2	2 (100%)	0	−	−	−	−
Quinine tablets	22	22 (100%)	0	22 (100%)	0	14 (62.5%)	8 (37.5%)
Quinine injections	6	6 (100%)	0	6 (100%)	0	−	−
Primaquine tablets	1	1 (100%)	0	1 (100%)	0	1 (100%)	0
Total	134	134 (100%)	0	132 (100%)	0	93 (74%)	33 (26%)

TLC = thin layer chromatography.

* 134 samples equals one adult dose (five were collected and Table 2 presents the number of individual tablets).

† Only tablets and capsules can be subjected to the disintegration test. At the time of this study, a MiniLab^®^ procedure for TLC for artemether syrup was not available (published in 2011).

**Table 4:** Drug information and results of quality tested by HPLC and dissolution

Drug name	Brand name (if applicable)	Strength (mg)	Pack size	Manufacturer	Country of manufacture	Batch number	Manufacturing date	Expiry date	No. of samples	Dissolution test (compliance with tolerance limits)	HPLC (contained stated amount of active ingredient)
Artesunate	Not stated	50	36	Aurochem Pharma	India	AFJ35A /08C01	03/2008	02/2011	2	Not undertaken	Yes
Artesunate	Falcigo	50	32	Zydus Adila Healthcare	India	ZHJ2175	06/2009	06/2011	1	Not undertaken	Yes
Sulfadoxine/Pyrimethamine	Fansidar	500/25	3	Efroze Chemical Industries	Pakistan	H003	07/2007	06/2010	2	No	Yes
Sulfadoxine/Pyrimethamine	Fansidar	500/25	14	Roche Pharm.	Pakistan	000267	01/2009	01/2012	2	No	Yes
Sulfadoxine/Pyrimethamine	Fansidar	500/25	14	Roche Pharm.	Pakistan	755	10/2005	10/2010	2	No	Yes
Sulfadoxine/Pyrimethamine	Fansidar	500/25	14	Roche Pharm.	Pakistan	P01075	02/2008	03/2011	2	No	Yes
Sulfadoxine/Pyrimethamine	Maladar	500/25	15	Roche Pharm.	Pakistan	P001103	01/2009	01/2013	1	No	Yes
Quinine	Howards	300	99	Howards	Pakistan	70321	05/2007	05/2012	1	No	Yes
Quinine	Howards	300	99	Howards	Pakistan	80576	08/2008	08/2013	1	No	Yes
Quinine	Howards	300	99	Howards	Pakistan	70322	05/2007	05/2012	1	No	Yes
Chloroquine	Not stated	150	30	Pars Darou	Iran	7800	12/2006	11/2010	1	Yes	Yes
Chloroquine	Not stated	150	30	Khawarazwy	Iran	n/a	07/2006	07/2010	1	Yes	Yes
Chloroquine	Not stated	150	30	Sanofi-Aventis	Pakistan	U060	05/2008	04/2012	1	Yes	Yes
Chloroquine	Nivaquine-P	250	30	Sanofi-Aventis	Pakistan	N165	12/2006	11/2010	2	Yes	Yes
Chloroquine	Nivaquine-P	250	30	Sanofi-Aventis	Pakistan	U067	06/2008	05/2012	2	Yes	Yes
Chloroquine	Nivaquine-P	250	30	Sanofi-Aventis	Pakistan	C014	01/2009	12/2012	1	Yes	Yes
Chloroquine	Nivaquine-P	250	30	Sanofi-Aventis	Pakistan	U007	01/2008	12/2011	1	Yes	Yes
Chloroquine	Nivaquine-P	250	30	Sanofi-Aventis	Pakistan	N165	12/2007	11/2011	1	Yes	Yes
Chloroquine	Nivaquine-P	250	30	Sanofi-Aventis	Pakistan	B178	09/2007	08/2011	1	Yes	Yes
Chloroquine	Nivaquine-P	250	30	Sanofi-Aventis	Pakistan	U067	06/2008	05/2012	1	Yes	Yes
Chloroquine	Nivaquine-P	250	30	Sanofi-Aventis	Pakistan	U195	06/2008	05/2012	1	Yes	Yes
Chloroquine	Nivaquine-P	250	30	Sanofi-Aventis	Pakistan	C049	02/2009	01/2013	1	Yes	Yes
Chloroquine	Nivaquine-P	250	30	Sanofi-Aventis	Pakistan	C098	02/2009	01/2013	1	Yes	Yes
Chloroquine	Nivaquine-P	250	30	Sanofi-Aventis	Pakistan	C085	04/2009	03/2013	1	Yes	Yes
Chloroquine	Nivaquine-P	250	30	Sanofi-Aventis	Pakistan	C014	12/2008	11/2012	2	Yes	Yes
Chloroquine	Nivaquine-P	250	30	Sanofi-Aventis	Pakistan	HC143	07/2009	06/2013	1	Yes	Yes
Chloroquine	Nivaquine-P	250	30	Sanofi-Aventis	Pakistan	C127	06/2009	05/2013	1	Yes	Yes
Chloroquine	Nivaquine-P	250	30	Sanofi-Aventis	Pakistan	B193	10/2007	09/2011	1	Yes	Yes
Chloroquine	Nivaquine-P	250	20	Sanofi-Aventis	Pakistan	U167	10/2008	09/2012	1	Yes	Yes
Chloroquine	Nivaquine-P	250	30	Sanofi-Aventis	Pakistan	U046	05/2008	04/2012	3	Yes	Yes
Complaint with USP tolerance limits	–	–	–	–	–	–	–	–	–	25/37 (68%)	40/40
Noncompliant with USP tolerance limits										12/37 (32%)	0
Total										37	40

HPLC = high-performance liquid chromatography; USP = U.S. Pharmacopeia.

Samples of chloroquine, sulfadoxine/pyrimethamine, and quinine were made by the same manufacturer but were from different batches as denoted by the batch number.
